# A nomogram to predict the cancer‐specific survival of stage II–IV Epithelial ovarian cancer after bulking surgery and chemotherapy

**DOI:** 10.1002/cam4.3980

**Published:** 2021-05-31

**Authors:** Ling Zhao, Ping Yu, Li Zhang

**Affiliations:** ^1^ Department of Gynecology Second Affiliated Hospital of Guizhou Medical University Qiandongnan Second People's Hospital Guizhou China; ^2^ Department of Gynecologic Oncology Dalian Medical University Dalian China; ^3^ Department of Obstetrics and Gynecology Northern Jiangsu People's Hospital Jiangsu China

**Keywords:** nomogram, ovarian cancer, prognosis, SEER, surgical therapy, survival

## Abstract

**Objective:**

In order to predict the survival rate of ovarian cancer patients, multiple independent risk factors are integrated to establish a prognostic nomogram.

**Methods:**

Cox analysis was used to construct the nomogram. All of the mainly independent factors, which can be used to predict 3‐year and 5‐year survival rates for cancer in the training cohort, were incorporated to establish nomograms. The C‐index, operating characteristic, ROC curves, and calibration plots can show evaluation results of performance.

**Results:**

Model derivation was based on 3277 patients who belong to different races. The best threshold for age was 51, 59, and 67 year old and the older the people, the worse their survival. Meanwhile, many lymph node examinations indicated a favorable survival and the survival of the positive set was worse than of that. In addition, the optional threshold was 64 mm for tumor size and the set larger than 64 mm had a better survival than that less than 64 mm. Univariate Cox proportional hazards regression model showed that the similar worse outcomes were showed in black race, advanced grade, stage T3, stage M1, lymph nodes positive, and CA125 positive compared with the first group. We found that the number of lymph nodes examined and tumor size had an inverse relationship with its corresponding score of CSS in training cases with bulking surgery and chemotherapy.

**Conclusions:**

We developed a model which relatively accurately predicted the prognosis of ovarian cancer by multiple univariate analysis, at the same time, the proposed nomograms exhibit superior prognostic discrimination and survival prediction.

## Graphical abstract

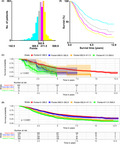

## 1 INTRODUCTION

Worldwide, ovarian cancer is one of the most common gynecological malignancies which remain asymptomatic diseases and the fifth most common female cancer.^1^ It was estimated that in 2018 around 300 thousand people will become new patients with ovarian cancer, and about 185 thousand people will die from ovarian cancer all over the world.^2^, ^3^ The treatment of ovarian cancer mainly depends on the International Federation of Gynecology and Obstetrics (FIGO) stage. Patients with ovarian cancer can be treated with more advanced medical techniques and drugs, such as more effective chemotherapeutic drugs,^4^ the improved cytoreductive surgery, radiotherapy, and targeted therapy,^5^ but the 5‐year relative survival rate (RSR) is still below 50%. Although the ovarian cancer mortality in developed countries has declined,^6^ this cancer is still the most common cause of cancer‐related death among gynecologic malignancies worldwide. In addition, studies have shown that age, race, and positive lymph nodes are important prognostic factors for ovarian cancer patients.^7^, ^8^, ^9^ Consequently, in order for doctors to make greater clinical decisions, benefit ovarian cancer patients, and enable individualized treatment and testing to be realized, accurate survival predictions need to be established urgently.

Nomogram is a simple visualization tool. In the field of oncology, this tool combines multiple variables to predict and quantify patient survival. Compared with the TNM staging system, the main feature of nomogram is individual prognosis of each patient. In terms of risk classification, personalized clinical management, and clinical trial design, the value of nomogram is significant. It is known that all exiting ovarian cancer nomograms are used for patients with localized disease, but there is not a special nomogram which is only used for ovarian cancer patients.^10^, ^11^ Therefore, in order to make up for this lack, in this article a prognostic nomogram and a risk stratification system for patients with ovarian cancer are established based on the database, Surveillance, Epidemiology, and End Results (SEER) database, which is a cancer database in the United States collecting information of 30% American population from 18 American registries.

We reviewed the diagnosis and treatment process of ovarian cancer patients in the past period, and analyzed some important basic information, surgical methods, and postoperative pathological classification. This article will establish a model to predict the prognosis of ovarian cancer by univariate analysis.

## 2 MATERIALS AND METHODS

### 2.1 Patients

From SEER‐18 database, information on newly diagnosed patients from 2004 to 2015 was obtained. The research objects in this article have the following characteristics: ovarian cancer patients with only one primary malignant tumor; bulking surgery and chemotherapy; without radiotherapy; stage II–IV; survival more than 3 months; epithelial ovarian cancer; follow‐up the completion date actively; clear clinicopathological information including age, gender, race, FIGO stage, therapy, etc. This study was not supervised by the Institutional Review Board, since data in SEER database are deidentified and available for the public. The following variables are related to every patients, such as TNM status, age, pathological subtype, race, histology grade, distant metastatic site, N stage, treatment strategy, T stage, vital status, as well as survival time.

In addition, TNM status in this article was redefined based on AJCC classification (2017 version).

### 2.2 Study population & data sources

Ovarian cancer cases studied in this article are from SEER program, which belongs to the largest national cooperation programs conducted by National Cancer Institute. The information about cancer incidence and survival for around 26% of American people is collected and published by this institute. The inclusion criterion for the current study was being pathologically confirmed with ovarian cancer from 2004 to 2015. We excluded patients not bulking surgery or chemotherapy, not stage II–IV, not epithelial ovarian cancer, not first tumor, with radiotherapy, survival less than 3 months, unknown CA125, unknown lymph nodes examined or positive, and unknown positive histology examination. Finally, information of 3277 ovarian cancer patients was used in this article. Information of 70% patients was used as the training cohort and information of other 30% patients was used as validation cohort, and all patients were assigned randomly.

### 2.3 Statistical analysis

Continuous variables that do not conform to the normal distribution are expressed as the median value (25th to 75th percentile), and other continuous variables that conform to the normal distribution are expressed as the mean and standard deviation (SD) values. Categorical variables are expressed as percentages. Chi‐square test and Student's *t*‐test are used to evaluate the differences in patient characteristics between two groups. Variables were also analyzed in multivariate Cox regression models if these variables were statistically significant in the univariate Cox regression models. Nomograms were constructed from the predictive model that includes identified prognostic factors. Prior to model inclusion, the variables were converted to the appropriate form based on the assumption of linearity. The optimal cut‐off point for continuous variables was determined using the X‐tile software.

The predictive accuracies of the constructed nomograms were evaluated using the concordance index (C‐index) as well as the area under the receiver operating characteristic curve varying with time (AUC).^12^ C‐index was first suggested by the professor, Frank E Harrell Jr who worked at Vanderbilt University in 1996, and is therefore also known as Harrell's concordance index. The main use of that index is to identify the discrimination degree between the predicted value from COX model and the reality in survival analysis. The most used at this stage is to predict the accuracy of the prognosis model of cancer patients. Through the calibration chart, we can assess the consistency between the predicted probability and the actual result. Calibration is related to how a model can provide unbiased estimates, and a plot on which predictive results fall along a 45◦ diagonal line can be got from a perfectly accurate nomogram. To evaluate a diagnostic method, ROC is only considered from the specificity and sensitivity, although accurate, patients do not necessarily benefit.

Statistical analyses were all performed using SPSS (version 22.0, SPSS, Chicago, IL, USA), R software (version 3.6.3; www.r‐project.org/), and X‐tile software (http://tissuearray.org/). Two‐sided *p* ≤ 0.05 indicates statistical significance.

## 3 RESULTS

### 3.1 Basic information of patients

**FIGURE 1:**
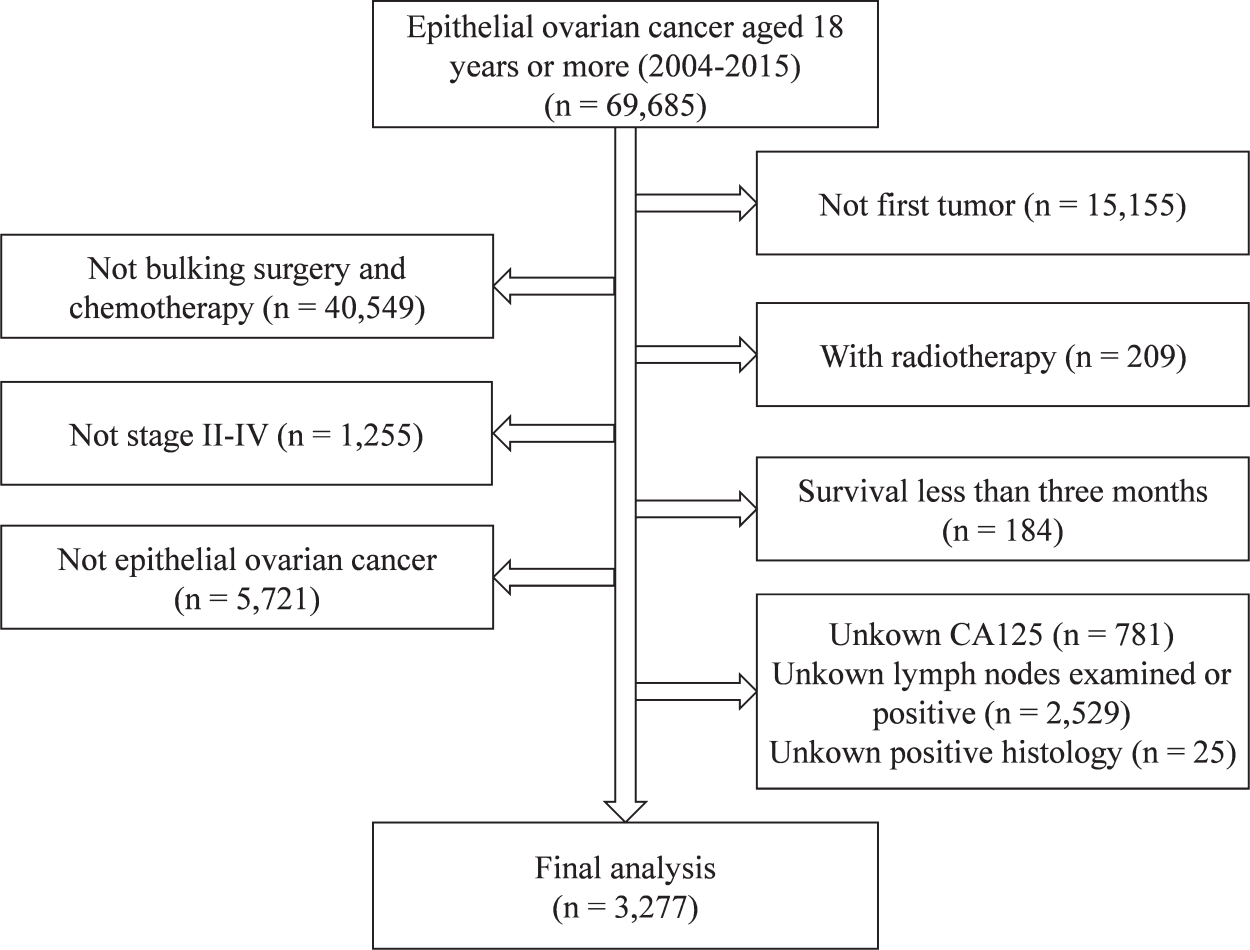
Data selection flowchart

The detailed data selection process was shown in Figure 1. Removing patients with unknown number of variables and not research objects, we finally got 3,277 cases. The patient's characteristics were listed in Table 1. Among the 3,277 cases identified in the database, there were 875 (26.7%) patients younger than 52 years old, 803 (24.5%) patients aged 52–59 years old, 848 (25.9%) patients with 60–67 years old, and 751 (22.9%) patients older than 68 years old. Moreover, we utilized R software to divide the figures into modeling group and verification group according to the ratio of 7:3. Only one significant difference in lymph nodes examined was found in the majority of comparisons. Most subjects were in stage III (66.7%), grade III & IV (86%), and serous histologic type (77.1%). This meant that these patients might be poorly differentiated and pathologically classified. In terms of diagnosis at lymph nodes, tumor size, and CA125, the proportion of lymph nodes positive (54.7%), larger than 65 mm (68.8%) and CA125 positive (95.8%) had the highest percentage. In contrast, less population with organ metastasis accounted for 23.0%.

**TABLE 1:** Basic clinical characteristics of ovarian cancer patients after bulking surgery and chemotherapy

Variables	Total	Training group	Testing group	*p* value
Age				
＜52	875(26.7%)	588(25.6%)	287(29.3%)	0.179
52–59	803(24.5%)	576(25.1%)	227(23.1%)	
60–67	848(25.9%)	601(26.2%)	247(25.2%)	
≥68	751(22.9%)	531(23.1%)	220(22.4%)	
Race				
White	2,803(85.5%)	1,968(85.7%)	835(85.1%)	0.438
Black	168(5.1%)	122(5.3%)	46(4.7%)	
Others	306(9.3%)	206(9.0%)	100(10.2%)	
Histological type				
Serous	2,525(77.1)	1,785(77.7%)	740(75.4%)	0.231
Endometrioid	185(5.6%)	133(5.8%)	52(5.3%)	
Mucinous	51(1.6%)	32(1.4%)	19(1.9%)	
Others	516(15.7%)	346(15.1%)	170(17.3%)	
Grade				
I	85(2.6%)	60(2.6%)	25(2.5%)	0.078
II	373(11.4%)	260(11.3%)	113(11.5%)	
III	1,636(49.9%)	1,178(51.3%)	458(46.7%)	
IV	1183(36.1%)	798(34.8%)	385(39.2%)	
Stage				
II	339(10.3%)	237(10.3%)	102(10.4%)	0.976
III	2,185(66.7%)	1,529(66.6%)	656(66.9%)	
IV	753(23.0%)	530(23.1%)	223(22.7%)	
T stage				
T1	43(1.3%)	27(1.2%)	16(1.6%)	0.544
T2	472(14.4%)	328(14.3%)	144(14.7%)	
T3	2,762(84.3%)	1,941(84.5%)	821(83.7%)	
N stage				
N0	1,454(44.4%)	1,026(44.7%)	428(43.6%)	0.577
N1	1,823(55.6%)	1,270(55.3%)	553(56.4%)	
M stage				
M0	2,524(77.0%)	1,766(76.9%)	758(77.3%)	0.827
M1	753(23.0%)	530(23.1%)	223(22.7%)	
Lymph nodes examined				
1–3	882(26.9%)	634(27.6%)	248(25.3%)	0.019*
4–9	792(24.2%)	540(23.5%)	252(25.7%)	
10–19	841(25.7%)	564(24.6%)	277(28.2%)	
≥20	762(23.3%)	558(24.3%)	204(20.8%)	
Lymph nodes positive				
Negative	1,483(45.3%)	1,048(45.6%)	435(44.3%)	0.493
Positive	1,794(54.7%)	1,248(54.4%)	546(55.7%)	
Tumor size(mm)				
＜65	1,022(31.2%)	725(31.6%)	297(30.3%)	0.461
≥65	2,255(68.8%)	1,571(68.4%)	684(69.7%)	
CA125				
Negative	139(4.2%)	95(4.1%)	44(4.5%)	0.651
Positive	3,138(95.8%)	2,201(95.9%)	937(95.5%)	

### 3.2 Analyze the impact of age, lymph nodes, and tumor size on survival

**FIGURE 2:**
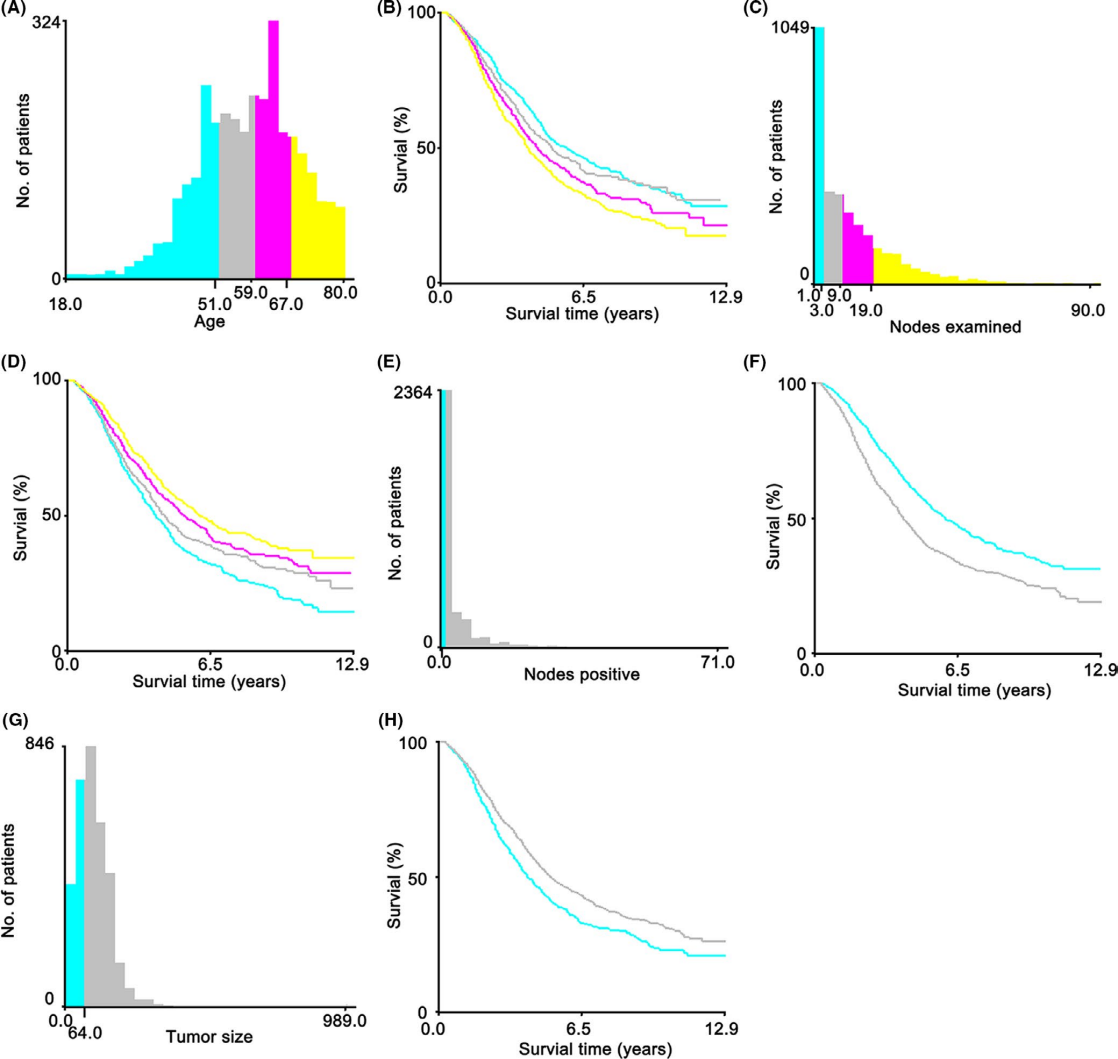
Identification of optimal cut‐off values of age (A, B), lymph nodes examined (C, D), lymph nodes positive (E, F) and tumor size (G, H) via X‐tile software analysis. Age could be divided into four groups: <52 years old, 52–59 years old, 60–67 years old, and ≥68 years old; the critical group for lymph nodes examined were 1–3 nodes, 4–9 nodes, 10–19 nodes, and ≥20 nodes; lymph nodes positive were divided into two categories, namely negative and positive; and the threshold in tumor size was 64 mm

To further investigate the role of age, lymph nodes, and tumor size on prognosis, we used X‐tile to present histogram of the data distribution and the optional cut‐off employing Kaplan–Meier curves. For age, the best threshold was 51, 59, and 67 year old and the older the people, the worse their survival (Figure 2AB). Meanwhile, lymph nodes examined were categorized into four subgroups: 1–3, 4–9, 10–19, and ≥20 (Figure 2C). It could be seen from the survival plot that more lymph node examinations indicated a favorable survival (Figure 2D). Lymph nodes were split in two groups (negative and positive group) and the survival of the positive set was worse than of that (Figure 2EF). In addition, the optional threshold was 64 mm for tumor size and the set larger than 64 mm had a better survival than that less than 64 mm (Figure 2GH).

### 3.3 Univariate and Multivariate analysis in training patients with EC after bulking surgery and chemotherapy

**FIGURE 3:**
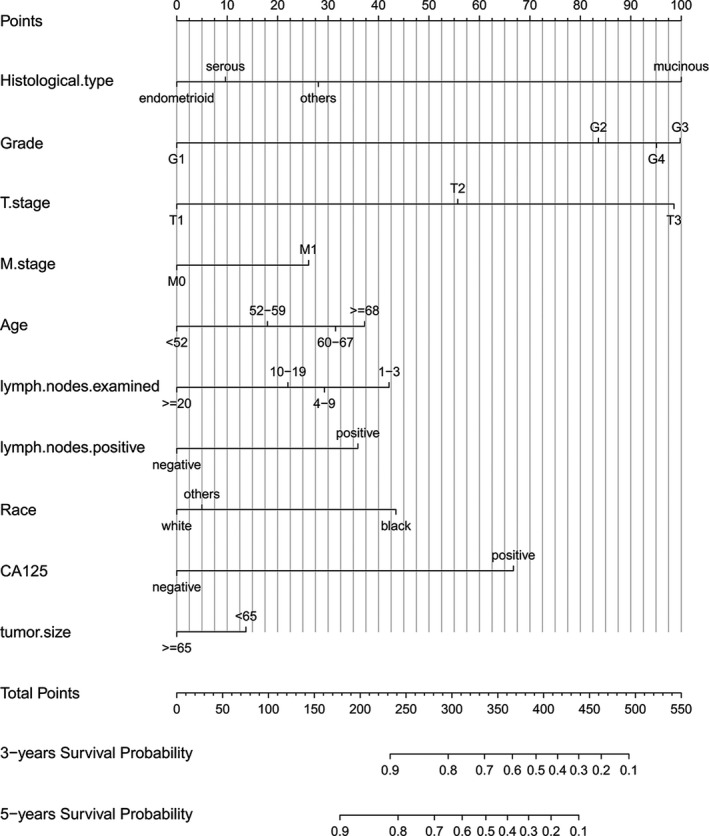
Nomograms to predict 3‐ and 5‐year cancer‐specific survival for patients with epithelial ovarian cancer after bulking surgery and chemotherapy

Univariate Cox proportional hazards regression model showed that age older than 68 years old harbored poorest prognosis (HR: 1.536; 95% CI: 1.298–1.818; *p *< 0.001) compared with other subjects. Additionally, the similar worse outcomes were showed in black race (HR: 1.586; 95% CI: 1.238–2.033; *p *< 0.001), advanced grade (HR: 3.054; 95% CI: 1.717–5.431; *p* < 0.001), stage T3 (HR: 2.003; 95% CI: 1.135–3.535; *p* = 0.017), stage M1 (HR: 1.675; 95% CI: 1.503–1.866; *p* < 0.001), lymph nodes positive (HR: 1.578; 95% CI: 1.398–1.781; *p* < 0.001), and CA125 positive (HR: 2.428; 95% CI: 1.620–3.640; *p* < 0.001) compared with the first group, while endometrioid EC patients (HR: 0.612; 95% CI: 0.457–0.819; *p* = 0.001), patients with examining lymph nodes number≥20 (HR: 0.594; 95% CI: 0.503–0.702; *p* < 0.001), and patients with tumor size ≥65 mm (HR: 0.782; 95% CI: 0.706–0.867; *p* < 0.001) decreased the probability of death in comparison to the first set. After normalization of the general characteristics of patients, including age, histology, race, M stage, grade, T stage, lymph nodes examined, lymph nodes positive, tumor size, and CA125, consistently, subjects older than 68 years old were revealed to harbor poorer prognosis (HR: 1.499; 95% CI: 1.304–1.724; *p* < 0.001) in comparison with others Figure 3. Furthermore, these mentioned factors were all confirmed to be independent prognostic indicators for cancer‐specific survival (CSS) in multivariate Cox analysis and these variables were selected to build a nomogram model. The detailed information was displayed in Table 2.

**TABLE 2:** Univariate and multivariable cox regression analysis of cancer‐specific survival

Variables	Univariate analysis		Multivariate analysis	
	HR	*p* value	HR	*p* value
Age				
＜52	Reference		Reference	
52–59	1.156(0.972–1.375)	0.101	1.190(1.030–1.375)	0.018
60–67	1.434(1.215–1.693)	＜0.001***	1.304(1.134–1.499)	＜0.001***
≥68	1.536(1.298–1.818)	＜0.001***	1.499(1.304–1.724)	＜0.001***
Race				
White	Reference		Reference	
Black	1.586(1.238–2.033)	＜0.001***	1.698(1.380–2.087)	＜0.001***
Others	1.044(0.845–1.289)	0.691	0.989(0.829–1.179)	0.899
Histological type				
Serous	Reference		Reference	
Endometrioid	0.612(0.457–0.819)	0.001**	0.874(0.683–1.118)	0.283
Mucinous	1.274(0.799–2.032)	0.31	2.542(1.725–3.746)	＜0.001***
Others	1.071(0.909–1.262)	0.411	1.213(1.059–1.390)	0.005**
Grade				
I	Reference		Reference	
II	2.391(1.320–4.330)	0.004**	2.374(1.487–3.791)	＜0.001***
III	3.225(1.822–5.710)	＜0.001***	2.690(1.715–4.221)	＜0.001***
IV	3.054(1.717–5.431)	＜0.001***	2.548(1.618–4.011)	＜0.001***
T stage				
T1	Reference		Reference	
T2	0.860(0.475–1.555)	0.617	1.086(0.598–1.974)	0.786
T3	2.003(1.135–3.535)	0.017*	1.964(1.108–3.482)	0.021*
M stage				
M0	Reference		Reference	
M1	1.675(1.503–1.866)	＜0.001***	1.436(1.285–1.604)	＜0.001***
Lymph nodes examined				
1–3	Reference		Reference	
4–9	0.839(0.716–0.983)	0.03*	0.879(0.770–1.003)	0.055
10–19	0.743(0.633–0.874)	＜0.001***	0.780(0.681–0.893)	＜0.001***
≥20	0.594(0.503–0.702)	＜0.001***	0.653(0.565–0.755)	＜0.001***
Lymph nodes positive				
Negative	Reference		Reference	
Positive	1.578(1.398–1.781)	＜0.001***	1.430(1.286–1.591)	＜0.001***
Tumor size(mm)				
＜65	Reference		Reference	
≥65	0.782(0.706–0.867)	＜0.001***	0.847(0.763–0.941)	0.002**
CA125				
Negative	Reference		Reference	
Positive	2.428(1.620–3.640)	＜0.001***	1.648(1.214–2.237)	0.001**

### 3.4 Construct a nomogram model of CSS in training cases with bulking surgery and chemotherapy

We made a nomogram model of CSS to detect the risk rate by significant factors among the training cohort. Each variable could be evaluated with a score from 0 to 100 and the corresponding sum of these scores ranging from 0 to 550 also assessed the corresponding 3‐year and 5‐year survival rate varying from 0.1% to 0.9%. Besides, exact score concerning each factor was presented in Table 3 and histological type and grade made a contribution to a highest point of 100. We found that the number of lymph nodes examined and tumor size had an inverse relationship with its corresponding score.

**TABLE 3:** The points of each variable

Characteristics	CSS nomogram
Age	
＜52	0
52–59	18
60–67	31.5
≥68	37.2
Race	
White	0
Black	43.5
Others	5.0
Histological type	
Serous	9.7
Endometrioid	0
Mucinous	100
Others	28.1
Grade	
I	0
II	83.6
III	99.8
IV	95.1
T stage	
T1	0
T2	55.7
T3	98.6
M stage	
M0	0
M1	26.1
Lymph nodes examined	
1–3	42.1
4–9	29.3
10–19	22
≥20	0
Lymph nodes positive	
Negative	0
Positive	35.9
Tumor size(mm)	
＜65	13.7
≥65	0
CA125	
Negative	0
Positive	66.7

### 3.5 Calibration chart between training set and validation set

**FIGURE 4:**
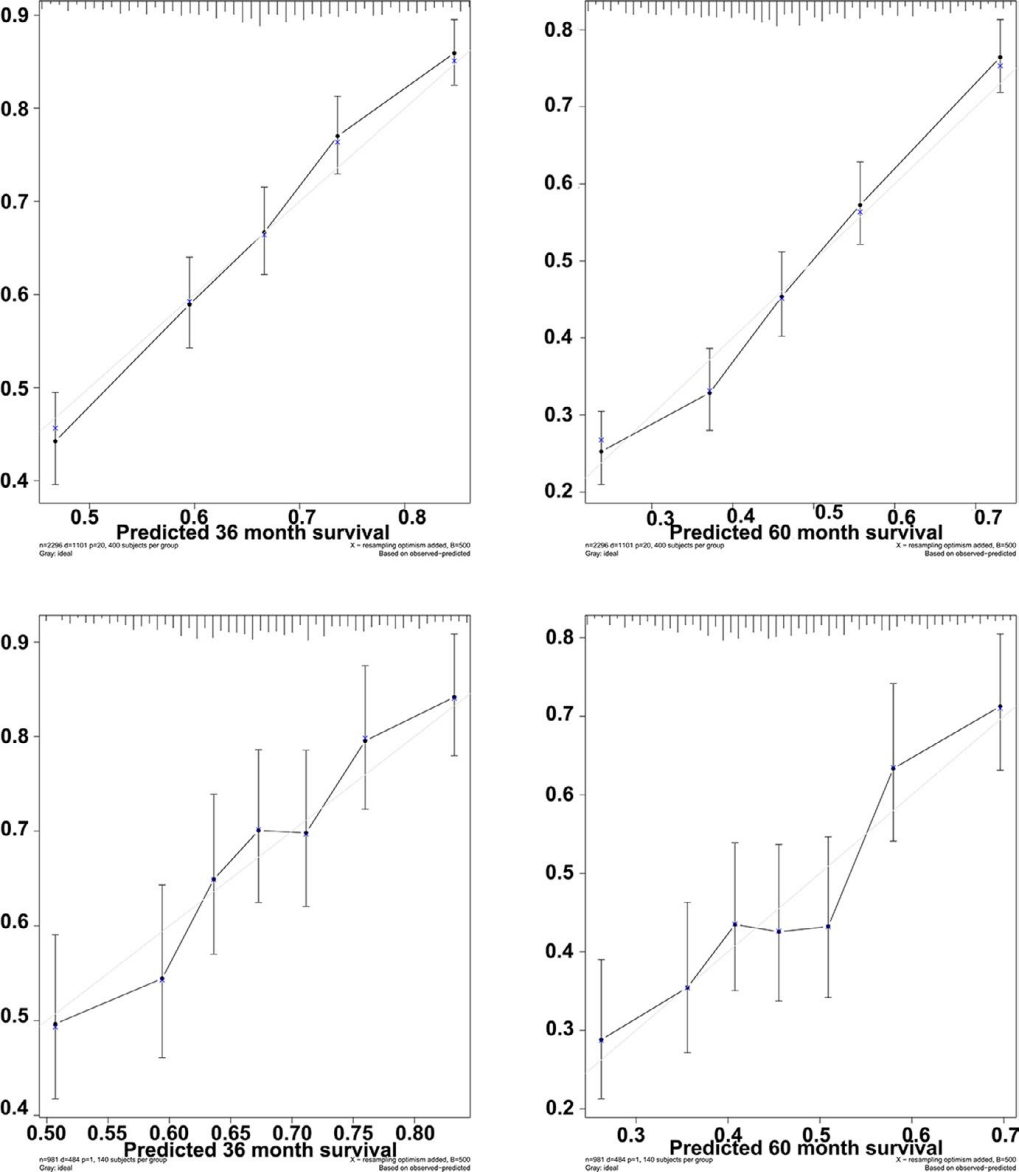
The calibration plot established for the nomogram in the training cohort and test cohort. x‐axis described nomogram‐predicted survival; y‐axis indicated observation survival. The graph along the 45‐degree line showed the ideal calibration model, where the predicted probability was consistent with the actual result. The two pictures above are the modeling group, and the two pictures below are the verification group

The c‐index was 0.66 in the modeling group and 0.647 in the verification group. The c‐index between two groups was so close. To further evaluate the consistency of the nomogram, we draw calibration plot to describe favorable prediction for 36‐month and 60‐month CSS in the modeling group and validation group (Figure 4).

### 3.6 Subgroup analysis of points in the nomogram

**FIGURE 5:**
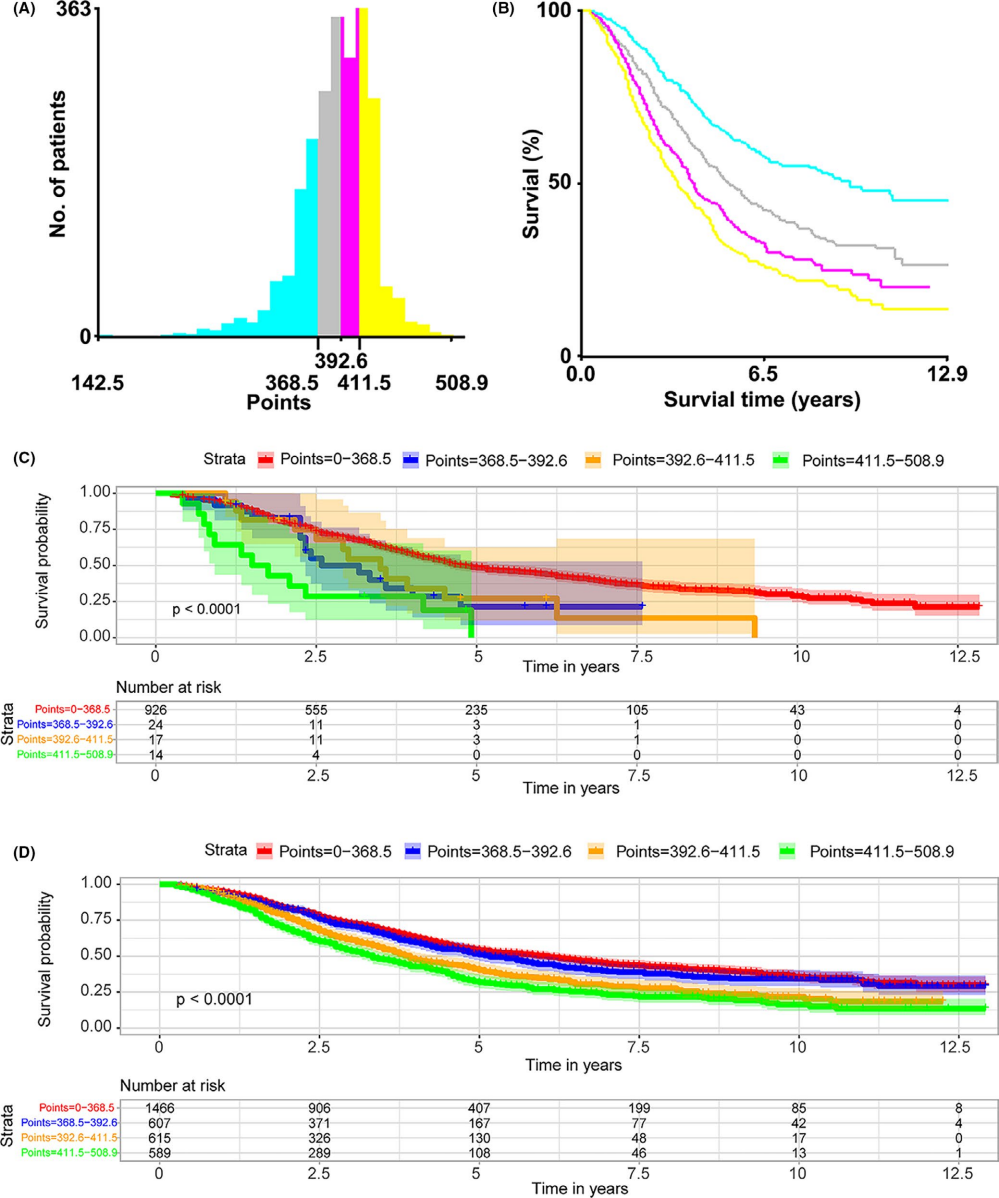
Kaplan–Meier survival curves of stratified risk groups in the training group, test group, and the whole cases. The risk scores were divided into four groups: 142.5–368.5 points, 368.6–392.6 points, 392.7–411.5 points, and ＞411.5 points

The X‐tile software was utilized to split the training patients into four subgroups according to total points. From Figure 5A, we could obtain the optimal cut‐off: −368.5, −392.6, and −411.5. Kaplan–Meier analysis was manifested with the survival curve of four subgroup which had statistically significant log‐rank *p* value. (Figure 5B). As a result, the similar method was applied in modeling set and all data (Figure 5CD). The definite *p* value of four group is shown in Table 4.

**TABLE 4:** The KM analysis for subgroup of points

Group	0–368.5	368.5–392.6	392.6–411.5	411.5–508.9
	(*p* value)	(*p* value)	(*p* value)	(*p* value)
Training group				
0–368.5		＜0.001***	＜0.001***	＜0.001***
368.5–392.6	＜0.001***		＜0.001***	＜0.001***
392.6–411.5	＜0.001***	＜0.001***		0.007**
411.5–508.9	＜0.001***	＜0.001***	0.007**	
Testing group				
0–368.5		0.022*	0.045*	＜0.001***
368.5–392.6	0.022*		0.764	0.097
392.6–411.5	0.045*	0.764		0.07
411.5–508.9	＜0.001***	0.097	0.07	
All data				
0–368.5		0.156	＜0.001***	＜0.001***
368.5–392.6	0.156		＜0.001***	＜0.001***
392.6–411.5	＜0.001***	＜0.001***		0.004**
411.5–508.9	＜0.001***	＜0.001***	0.004**	

## 4 DISCUSSION

In our research, the real information of patients with ovarian cancer after major surgery and chemotherapy is used in the research. In addition, based on the SEER database, a prognostic nomogram and risk stratification system were established by us. Otherwise, the nomogram exhibits excellent performance both internally and externally, which is consistent with the results of the calibration, C‐index, and ROC curve.

We found few studies focused on prediction of the cancer‐specific survival of stage II–IV epithelial ovarian cancer after bulking surgery and chemotherapy. Two main reasons can be used to explain why this study focused on the nomogram of the cancer‐specific survival of stage II–IV epithelial ovarian cancer after bulking surgery and chemotherapy. First, ovarian cancer is high heterogeneity with extremely poor survival, and the 5‐year survival was less than 30%, however, different patients showed various prognosis of ovarian cancer. It is difficult to conduct individualized clinical management and surveillance, since the lack of a reliable model which can predict survival in ovarian cancer. Second, given the fact that stage II–IV epithelial ovarian cancer patients after bulking surgery and chemotherapy are characterized by significantly increased incidence and mortality rate, confounding bias in general prognostic indicators may occur.

In the United States, ovarian cancer mortality in Hispanic patients varied by sub‐ethnicity.^13^ Compared with other races, white women are more likely to get epithelial ovarian cancer, while African American women with this cancer have a higher mortality rate.^14^ In the short‐term survival period, age, stage, marital status, ethnicity/race, and surgery were all more strongly related to mortality.^15^ However, compared with the United States, in Japan epithelial ovarian cancer shows remarkably different characteristics. The number of clear cell carcinoma has shown a dramatic rise which accounts for about 30% epithelial ovarian cancer between 2002 and 2015 in Japan.^16^ Within 2 years after diagnosis, clear cell carcinoma and carcinosarcoma show a higher risk of death than high‐grade serous.^17^ In the localized and regional stages, carcinosarcoma and malignant Brenner tumors exhibit the highest mortality rate, while clear cell and carcinosarcoma have the worst prognosis in distance stage.^18^

In this study, we used the exclusion criteria for screening, univariate analysis and multivariate analysis of 3277 patients in the SEER‐18 database. And, we used SEER Stat software version 8.3.5 to extract information of these patients from 2004 to 2015. Exclusion criteria included not bulking surgery and chemotherapy, not stage II–IV, not epithelial ovarian cancer, not first tumor, with radiotherapy, survival less than 3 months, unknown CA125, unknown lymph nodes examined or positive, and unknown positive histology examination. Statistical model based on a large number of pathological data of clinical surgery patients was used by Lei et.al to find the optimal LNs which pT stages can examine and stratify for EOC patients.^19^ For stage I and IV, it is not clear to classify LNR positives into three categories.^20^ The critical group for lymph nodes examined were 1–3 nodes, 4–9 nodes, 10–19 nodes, and ≥20 nodes; 1–3 nodes and positive lymph nodes are more meaningful in new model.

There were 10 independent prognostic variables for CSS, including histological type, T stage, grade, M stage, age, lymph nodes examined, lymph nodes positive, race, CA125, and tumor size. Identification of optimal cut‐off values of age range (A, B), lymph nodes examined (C, D), lymph nodes positive (E, F), and tumor size (G, H) via X‐tile software analysis. Age could be divided into four groups: <52 years old, 52–59 years old, 60–67 years old, and ≥68 years old; the critical group for lymph nodes examined were 1–3 nodes, 4–9 nodes, 10–19 nodes, and ≥20 nodes; lymph nodes positive were divided into two categories, namely negative and positive; and the threshold in tumor size was 64 mm. In multivariate analysis, given the fact that histological type and grade had been regarded as significant independent factors. Compared with nine variables, histological type showed the greatest discriminating power. Grade and T stage are the next important factors, which depend on the pathology after surgery. Intriguingly, tumor size was largely negative than others, which is highly related to the symptom. In this study, the importance of CA125 was once again affirmed as an independent and easily obtained indicator before surgery, and it was significantly positively correlated with the risk of death of ovarian cancer patients. Additionally, as we identified using 981 patients，30% randomly selected patients from total patients, performed external verification and grade and lymph nodes examined showed good predictive ability.

Finally, we used calibration plot to demonstrate the clinical effectiveness of our nomograms and made Kaplan–Meier survival curves, whose risk scores were divided into four groups: 142.5–368.5 points, 368.6–392.6 points, 392.7–411.5 points, and ＞411.5 points. High predictive accuracy is more likely to translate into usefulness in clinical practice. When applied to the training and validation groups, the nomograms developed in this study consistently achieved good predictive accuracy, reliability, and repeatability, so high‐risk patients with good survival times assessed according to current standards can be detected by the new model. Additionally, the use of this model is helpful to reduce the heterogeneity between different treatment groups, because the stratification of patients with ovarian cancer based on the predicted prognosis has been implemented by the model in clinical trials. Especially, preliminary studies were performed analyzing in our modle development. The calibration plot established for the nomogram in the training cohort and test cohort (Figure 4). x‐axis described nomogram‐predicted survival; y‐axis indicated observation survival. The graph along the 45‐degree line showed the ideal calibration model, where the predicted probability was consistent with the actual result. If the threshold probability of the net benefit is impractical, a well‐performing model may have limited applicability. A prognostic nomogram utilizes an elegant graphical interface to provide a simplified representation of a complicated statistical model. Compared with other predictive models, nomograms are more accurate and comprehensible, and their user‐friendly interface allows their wide application in clinical practice.

As such, this new model can be used to identify high‐risk patients who had stage II–IV epithelial ovarian cancer after bulking surgery and chemotherapy.

## 5 LIMITATION

First, this study may have selection bias, because patients studied are these the expected survival rate can be calculated using a nomogram, and patients who do not meet the conditions were excluded. Second, the predicted value from nomograms is only reference information for clinicians, not an accurate prognosis. Third, the results are only for patients in the United States, and cannot be representative of other countries, because only patients in the United States were removed for verification of the nomogram and risk segmentation. Therefore, external verification in different countries is needed. Besides, the gold standard for validating nomogram performance is to use it in patients in randomized clinical trials. To sum up, the first nomogram and risk stratification system were built in our study for patients initially diagnosed with ovarian cancer to their OS prediction. The model showed great performance and clinical utility in internal and external verification, but further evaluation in other independent groups is necessary.

## CONFLICT OF INTEREST

The authors have no conflict of interest to declare.

## Data Availability

All authors declaimed that data availability. Ethics approval: as the study consisted of the retrorespective analysis of anonymized data according to the local ethics committee, a special approval is generally not required.
